# *ACTA1* is inhibited by *PAX3-FOXO1* through RhoA-MKL1-SRF signaling pathway and impairs cell proliferation, migration and tumor growth in *Alveolar Rhabdomyosarcoma*

**DOI:** 10.1186/s13578-021-00534-3

**Published:** 2021-01-28

**Authors:** Qiande Hu, Liang Zhu, Yuan Li, Jianjun Zhou, Jun Xu

**Affiliations:** 1 Research Center for Translational Medicine, Shanghai East Hospital, Tongji University School of Medicine, 150 Jimo Road, Shanghai, 200120 China; 2 Shanghai East Hospital, Tongji University School of Medicine, Shanghai, 200120 China

**Keywords:** *ACTA1*, *PAX3-FOXO1*, *Alveolar rhabdomyosarcoma*, RhoA-MKL1-SRF signaling pathway, Cell proliferation, Tumor growth

## Abstract

**Background:**

*Alveolar Rhabdomyosarcoma* (ARMS) is a pediatric malignant soft tissue tumor with skeletal muscle phenotype. Little work about skeletal muscle proteins in ARMS was reported. *PAX3-FOXO1* is a specific fusion gene generated from the chromosomal translocation t (2;13) (q35; q14) in most ARMS. *ACTA1* is the skeletal muscle alpha actin gene whose transcript was detected in ARMS. However, *ACTA1* expression and regulation in ARMS have not been well investigated. This work aims to explore the expression, regulation and potential role of *ACTA1* in ARMS.

**Results:**

ACTA1 protein was detected in the studied RH30, RH4 and RH41 ARMS cells. *ACTA1* was found to be inhibited by *PAX3-FOXO1* at transcription and protein levels by employing western blot, luciferase reporter, qRT-PCR and immunofluorescence assays. The activities of *ACTA1* gene reporter induced by RhoA, MKL1, SRF, STARS or Cytochalasin D molecule were reduced in the presence of overexpressed PAX3-FOXO1 protein. CCG-1423 is an inhibitor of RhoA-MKL1-SRF signaling, we observed there was a synergistic effect between this inhibitor and *PAX3-FOXO1* to suppress *ACTA1* reporter activity. Furthermore, *PAX3-FOXO1* overexpression decreased *ACTA1* protein level and knockdown of *PAX3-FOXO1* by siRNA enhanced *ACTA1* expression. In addition, both MKL1 and SRF, but not RhoA were also found to be inhibited by *PAX3-FOXO1* gene at protein levels and increased once knockdown of *PAX3-FOXO1* expression. The association between MKL1 and SRF in cells was decreased accordingly with ectopic expression of PAX3-FOXO1. However, the distribution of MKL1 and SRF in nuclear or cytoplasm fraction was not changed by *PAX3-FOXO1* expression. Finally, we showed that *ACTA1* overexpression in RH30 cells could inhibit cell proliferation and migration in vitro and impair tumor growth in vivo compared with the control groups.

**Conclusions:**

*ACTA1* is inhibited by *PAX3-FOXO1* at transcription and protein levels through RhoA-MKL1-SRF signaling pathway and this inhibition may partially contribute to the tumorigenesis and development of ARMS. Our findings improved the understanding of *PAX3-FOXO1* in ARMS and provided a potential strategy for the treatment of ARMS in future.

## Introduction

*Rhabdomyosarcoma* (RMS) is the most common soft tissue tumor in children and young adults with an incidence of about six cases per 1,000,000 population per year [1, 2]. *Embryonal Rhabdomyosarcoma* (ERMS) and *Alveolar Rhabdomyosarcoma* (ARMS) are the two major morphologic subtypes of RMS characterized on the basis of their clinical and histopathological features [3, 4]. ERMS is more common and favorable to treatment than ARMS. In contrast, ARMS is more aggressive and has a worse outcome than ERMS. Specifically, most ARMS are characterized by chromosomal translocation of either t (2; 13) (q35; q14) or t (1; 13) (q36; q14), mainly generating *PAX3-FOXO1* and *PAX7-FOXO1* fusion genes, respectively. These fusion genes encode chimeric proteins PAX3/7-FOXO1, which consist of the N-terminal DNA-binding domain of PAX3/7 and the C-terminus of the transactivation domain of FOXO1 protein [5, 6]. Both PAX3-FOXO1 and PAX7-FOXO1 are expressed at higher levels and have more potent transcriptional activities than the wild types of PAX3/7 proteins in ARMS tumors. But *PAX3-FOXO1* is more common accounting for about 55% of ARMS cases than *PAX7-FOXO1* with 22% of ARMS and is associated with worse prognosis and lower overall survival rate in this disease [7–9].

Numerous studies have shown that *PAX3-FOXO1* is oncogenic and involved in ARMS tumorigenesis [10–12]. Exogenous expression of *PAX3-FOXO1* could cause the transformation of chicken embryo fibroblast cells to become enlarged and grow in multiple layers [10]. In the study using immortalized human myoblast, cells expressing PAX3-FOXO1 protein developed tumor in immunocompromised mice [11]. Knockdown of *PAX3-FOXO1* expression by siRNA oligonucleotide in ARMS cells reduced the cell motility, inhibited the rate of cellular proliferation and induced the muscle differentiation [12]. However, the detailed mechanism of *PAX3-FOXO1* implicated in ARMS tumorigenesis is still not fully understood.

Skeletal muscle alpha-actin protein (ACTA1), encoded by *ACTA1* gene, belongs to the actin protein family consisting of six isoforms in human [13]. ACTA1 isoform is the major component in skeletal muscle thin filament of sarcomere and is essential for force production, muscle contraction and movement [13, 14]. *ACTA1* expression is developmentally and transcriptionally regulated in vivo. In chicken skeletal muscle, vascular actin (ACTA2) is the first muscle actin to be expressed in the myotome, then ACTA2 is downregulated and cardiac actin (ACTC) expression increases. At the time of birth, cardiac actin expression is downregulated and ACTA1 expression is increased and remains the major isoform in adult skeletal muscle [15, 16]. A similar developmental process occurs for *ACTA1* in human skeletal muscle [17, 18]. At the transcriptional level, *ACTA1* expression is mainly modulated by serum response factor (SRF) [19, 20]. SRF is a MADS-box transcription factor that is highly conserved and ubiquitously expressed and can regulate muscle-specific gene expression by binding to the CC(A/T)_6_GG consensus sequence (also called CArG box) within the promoter region of target genes [21]. SRF controls *ACTA1* transcription and expression by binding to CArG box and associating with the coactivator myocardin-related transcription factor A (MRTF-A/MKL1/Mal/BSAC). MKL1 is one member of the MRTF family which consists of myocardin, MKL1 and MKL2. MKL1 acts as a cofactor to associate with SRF and stimulate SRF-dependent target gene transcription. MKL1 activity is modulated by actin dynamics. MKL1 is localized to the cytoplasm by directly binding to monomeric globular-actin (G-actin) through the N-terminal RPEL domains, but once actin polymerization to form filamentous actin (F-actin) occurs in response to Rho signaling, MKL1 translocalizes into and accumulates in nucleus, where it activates the transcription of SRF target genes such as *ACTA1* [22–24].

Being a structural component of skeletal muscle, *ACTA1* is also implicated in a variety of muscle diseases. *ACTA1* knockout in mice causes muscle weakness and death in the early neonatal period [15, 25]. Amino acid mutations in ACTA1 protein are responsible for the congenital myopathies with muscle weakness such as nemaline myopathy (NM), intranuclear rod myopathy (IRM) and actin myopathy (AM) [13]. However, few studies have been reported about the behavior of *ACTA1* in cancer disease, especially in ARMS.

Here, we firstly examined *ACTA1* expression and found that *ACTA1* was inhibited by *PAX3-FOXO1* in ARMS cells. We later analyzed the detailed mechanism and showed that RhoA-MKL1-SRF signaling was involved in this *ACTA1* inhibition by *PAX3-FOXO1* in ARMS cells. Finally, we determined the potential role or function of *ACTA1* in ARMS and the in vitro and xenograft assays showed that *ACTA1* overexpression could suppress cell proliferation and tumor growth. Therefore, our data provide a new insight to further understand the tumorigenesis or progression of ARMS and a potential strategy for ARMS treatment or prognosis in future.

## Materials and methods

### Cell culture

Human *Alveolar Rhabdomyosarcoma* cell lines RH30(SJ-RH30), RH4(SJ-RH4) and RH41(SJ-RH41) were generously provided by Dr. Grosveld GC (St. Jude Children's Research Hospital, USA) and cultured in DMEM (Hyclone) supplemented with 10% fetal bovine serum, 100 U/ml penicillin, and 100 ug/ml streptomycin at 37 ℃ in a 5% CO_2_ atmosphere. All these cell lines were authenticated via STR profiling by BCPCA (Beijing, China) and confirmed by western blot analysis for PAX3-FOXO1 expression. RH30/vector and RH30/*ACTA1* (RH30 cells stably transfected with p CMV-Tag-2B vector or human *ACTA1* expression plasmid, respectively) were maintained in the same DMEM as untransfected ARMS cells but supplemented with geneticin (200 µg/ml).

### Plasmids and reagents

*PAX3-FOXO1/FKHR* expression plasmid was described previously [26]. Serum response factor (SRF) and RhoA (Q63L) expression plasmids were from the stock in our lab. MKL1 expression plasmid was a gift of Dr. Prywes R. (Columbia University, USA). Human *STARS* and *ACTA1* expression plasmids were individually constructed by inserting the *STARS*, *ACTA1* ORF (open reading frame) from RH30 cDNA into p CMV-Tag-2B (Stratagene) vector at the BamHI and PstI sites. The *ACTA1* reporter plasmid (546*ACTA1*) was constructed by cloning the human *ACTA1* promoter region (−464 to + 82 bp) into p Luc-MCS vector (Stratagene) using the forward primer 5′-tagAGATCTatctgagcaaagaacccgaag-3′ and reverse primer 5′-agtGAGCTCtagctacaactgctactctcggct-3′ (BglII and SacI sites are shown in uppercase letters) for PCR amplification. 546*ACTA1* deletion mutants were made by PCR amplifications, primers used: 5′-ataAGATCTcgctagggagacactccata-3′ (forward) for 321*ACTA1*; 5′-ataAGATCTccaggccgcgaaccggccga-3′ (forward) for 224*ACTA1* and 5′-tatAGATCTcagcgacattcctgcggggt-3′ (forward) for 158*ACTA1*. The common reverse primer for 546*ACTA1* mutants is 5′-actGAGCTCttaccaacagtaccggaatg-3′.

All the constructs were verified by sequencing. Antibodies used were: anti-ACTA1 (No.17521–1-AP, Proteintech). anti-FOXO1 (No. 2880, Cell Signaling Technology). anti-Flag (No. F1804, Sigma). anti-MKL1 (No. HPA030782, Sigma). anti-SRF (No. SC-25290, Santa Cruz Biotechnology). anti-GAPDH (No. ab181602, Abcam). anti-TBP (No. A10185, Abclonal). anti-ɑ-Tubulin (No. ab28037, Abcam) and anti-SRF (No. 5147, Cell Signaling Technology). CCG-1423 was purchased from MedChem Express. Cytochalasin D was obtained from Gene Operation.

### Luciferase reporter assay

Human *Alveolar Rhabdomyosarcoma* cells RH30, RH4 or RH41 were seeded in 24-well plate at a density of 0.5–1.0 × 10^5^ cells in 0.5 ml DMEM antibiotic-free growth medium and transiently transfected using lipofectamine 2000 reagent according to the manufacture’s instruction. A total of 0.35–0.40 ug plasmids DNA per well with 50 ng reporter, 2 ng renilla luciferase plasmid (pRL-TK internal control, Promega) and 300–350 ng *PAX3-FKHR* or other indicated expression plasmids were used for each transfection. The empty vector pcDNA3.1 or pCMV-Tag-2B was used to keep equal amount of total plasmid in transfection. Cells were placed in growth medium overnight after transfection and then serum free-starved (DMEM-0.3%FBS) for 24-36 h before luciferase activity was analyzed. Firefly luciferase activity was measured using Dual-Luciferase assay kit (Promega) with a luminometer (Lumat LB 9507, Berthold Technologies) and normalized to renilla luciferase activity. The activity difference was expressed as the fold change compared to the activity obtained from empty vector control that was set as 1. All assays were performed in duplicate and repeated independently at least three times. The error bar indicated the standard error of the mean (SEM) of the data from duplicate samples assayed.

### Immunofluorescence assay

Cells grown on glass coverslips in 24-well plate were transiently transfetcted with empty vector, MKL1 and/or *PAX3-FOXO1* using Lipofectamine reagent. Cells were maintained in DMEM-0.3% FBS for 24 h after transfection. For staining, cells were washed for two times with PBS and fixed in 4% paraformaldehyde/PBS for 20 min at room temperature, then blocked with 3% donkey serum/0.3% Triton X-100/0.05%Tween-20/PBS for 1 h, followed by incubation with primary antibodies at 4 °C overnight. Coverslips were subsequently incubated with Alexa Fluor 488- and/or Alexa Fluor 633-conjugated donkey anti-mouse or donkey anti-rabbit secondary antibody (1:1000 dilution, Biotium) for 1 h at room temperature. The nuclear DNA was stained using DAPI in PBS for 10 min. Cells were observed and imaged under a Leica TCS sp5 confocal scanning laser microscope (Leica Laser Technik, GmmbH, Germany).

### Western blot analysis

Cells transfected with increasing amount of *PAX3-FOXO1* expression plasmid were harvested and lysed in RIPA buffer (Beyotime, Jiangsu, PRC) containing protease inhibitor cocktail (Thermo scientific) and 1 mM phenylmethylsulfonyl fluoride (PMSF). The cell lysates were centrifuged at 12,000 rpm for 20 min at 4 ℃ and the protein concentrations were quantified by BCA assay kit (Beyotime, Jiangsu, PRC). The nuclear and cytoplasmic proteins from the transfected cells were obtained by nuclear and cytoplasmic protein extraction kit according to the manufacturer instructions (Beyotime, Jiangsu, PRC). A total of 50 to100 ug/lane of proteins were separated by 10% SDS-PAGE and electroblotted to nitrocellulose or polyvinylidene difluoride (PVDF) membrane. The membrane was blocked with 5% non-fat milk-TBST, sequentially incubated with primary antibody overnight at 4 ℃ and horseradish peroxidase-conjugated secondary antibody for 1 h at room temperature. Protein bands were detected by use of an enhanced chemiluminescent substrate (Thermo scientific). The digital chemiluminescent images (blot images) were captured by a GE LAS 4000 chemiluminescence imager. The densities of protein bands were quantified using the ImageJ software.

### qRT-PCR analysis

Total RNA was extracted from the RH30 cells transfected with expression plasmids *PAX3-FOXO1,* MKL1 or both using Trizol reagent following the manufacture’s instruction. One microgram of total RNA was used for complementary DNA synthesis using Takara RT–PCR kit (Takara Biotechnology, Dalian, PRC) and random hexamer primers. mRNA levels were analyzed by quantitative Real-Time PCR (qRT-PCR) in 20 µl volume containing 25 to 50 ng cDNA using SYBR Premix Ex Taq II kit (TaKaRa) and an ABI 7500 Real Time PCR Detection System (Applied Biosystems). Primers used for qRT-PCR were: 5′-GGCATTCACGAGACCACCTAC-3′ (forward) and 5′-CGACATGACGTTGTTGGCATAC-3′ (reverse) for *ACTA1,* 5′-CCTCTCACCTCAGAATTCAATT-3′ (forward) and 5′-TCTGGATTGAGCATCCACCAAG-3′ (reverse) for *PAX3-FOXO1* and 5′-CTGGGCTACACTGAGCACC-3′ (forward) and 5′- AAGTGGTCGTTGAGGGCAATG-3′ (reverse) for *GAPDH* as an internal control for normalization. PCR cycling conditions were set to 95 °C for 30 s, followed by 40 cycles at 95 °C for 5 s, and 60 °C for 34 s. Relative expression level of *ACTA1*was calculated according to the comparative 2^− △△Ct^ method.

### Knockdown of *PAX3-FOXO1 *by small interfering RNAs (siRNAs)

The following siRNAs targeting *PAX3-FOXO1* and non-targeting control (NTC) were synthesized by Genomeditech Co., Ltd (Shanghai, PRC) according to the sequences described in [12]. The siRNAs SiPF2 (sense: 5′-CCUCUCACCUCAGAAUUCAdTdT-3′; antisense: 5′-UGAAUUCUGAGGUGAGAGGdTdT-3′) and siPF3 (sense: 5′-CUCUCACCUCAGAAUUCAAdTdT-3′; antisense: 5′-UUGAAUUCUGAGGUGAGAGdTdT-3′) were for *PAX3-FOXO1*. And the siCON (sense: 5′-CUACUAUACCGAUACUCCCdTdT-3′; antisense: 5′-GGGAGUAUCGGUAUAGUAGdTdT-3′) was used as the NTC siRNA. The RH41 or RH30 cells were transfected with siRNAs at a final concentration of 20 nM by lipofectamine 2000 and kept in serum free DMEM for about 60 h, then harvested for protein expression analysis.

### CCK-8 cell viability analysis

RH30 cells stably transduced with *ACTA1* (RH30/ *ACTA1*) or empty vector (RH30/ vector) were plated in triplicate in 96-well plate at 2 × 10^3^ /well in 100 ul DMEM supplemented with 10% FBS and cultured under normal conditions with a 5% CO_2_ atmosphere at 37 ℃. At different time points (0 h, 12 h, 24 h, 36 h, 48 h and 72 h), cell viability was measured by the Cell Counting Kit-8 (CCK-8, Sangon Biotech, Shanghai, PRC). The optical density (OD) value of each well was determined at 450 nm by a microplate reader (SpectraMax M5, Molecular Devices).

### In vitro wound healing assay

Stable cell line RH30/*ACTA1* or control RH30/ vector was seeded in 6-well plate at about 3 × 10^5^ cells per well in DMEM growth medium. When the cells grew to 90% confluence and formed monolayer, a scratch was made to across the center of the well with a 200 µl sterile pipette tip. The cells were gently washed twice with PBS to remove the floating cells and incubated in serum-free DMEM for 24–48 h. The movements of the cells into the scratch were photographed on a microscope at 0 h, 24 h and 48 h, respectively. The average migration distance was measured using ImageJ software.

### Transwell migration assay

The in vitro transwell migration was performed by culturing the stable cell line Rh30/*ACTA1* or RH30/vector control into the upper insert chamber in 24-well plate (filter with 8-µm pore, Corning Costar, MA, USA) at 4 × 10^4^ cells in 200 ul serum free DMEM medium. 600 µl DMEM with 20% FBS was added to the lower chamber. After 36-48 h incubation at 37 ℃, cells remaining in the upper chamber were removed by cotton swab and cells that migrate onto the lower surface of the filter were fixed with 4% with paraformaldehyde (PFA), stained with 0.3% crystal violet for 30 min and counted in 3–5 different fields under an inverted microscope (100 × magnification).

### In vivo tumor growth assay

Tumor growth assay was performed using 5–8-week-old male athymic BALB/c mice purchased from Shanghai Slac Laboratory Animal Co. Ltd. (Shanghai, PRC). The mice were maintained in a specific pathogen-free animal care facility at the Tongji University Animal Experimental Center. Briefly, RH30/vector control and RH30/*ACTA1* stable cells were trypsinized, counted and resuspended in PBS. Then 2 × 10^6^ cells in 200 ul PBS were subcutaneously injected into the hind limbs of the mice. Total ten mice were used, and each was injected with RH30/vector control in the left flank and RH30/*ACTA1* in the right flank of hind legs, respectively. Tumor growth was monitored every other day and the tumor dimension was measured using a digital caliper two times a week. The tumor volume was calculated according to the formula V = 0.52 × L × W^2^ where the L and W represent the length and width of the tumor, respectively. At the end of the experiment, the mice were sacrificed and the tumors were harvested, frozen and stored at − 80 ℃ for further analysis. All the mouse work was carried out according to the protocols approved by the Committee on the Ethics of Animal Experiments of Tongji University.

### Tumor immunohistochemistry analysis

These frozen sections from xenograft tumor samples derived from RH30/vector and RH30/*ACTA1* cells were cut in 7–15 µm thick by a cryostat microbiotom and analysed by immunohistochemistry following the standard IHC staining procedures. The antibodies used for staining were Ki67 (ER1706-46, 1:400, Hubio, PRC) and Flag (0912–3, 1:50, Hubio, PRC). H&E staining was used to assess cell morphology. The stained slides were imaged and evaluated by the experienced pathologists at Shanghai East Hospital and the Service Center in Huabio Company, respectively.

### Statistical analysis

Experiments were repeated at least three times and data were expressed as mean ± SEM. Difference between two groups was analyzed by two-tailed Student’s t test. Difference with P < 0.05 (*) or P < 0.01 (**) was considered statistically significant.

## Results

### Protein expression of *ACTA1* in *Alveolar Rhabdomyosarcoma* (ARMS) cells

**Fig. 1:**
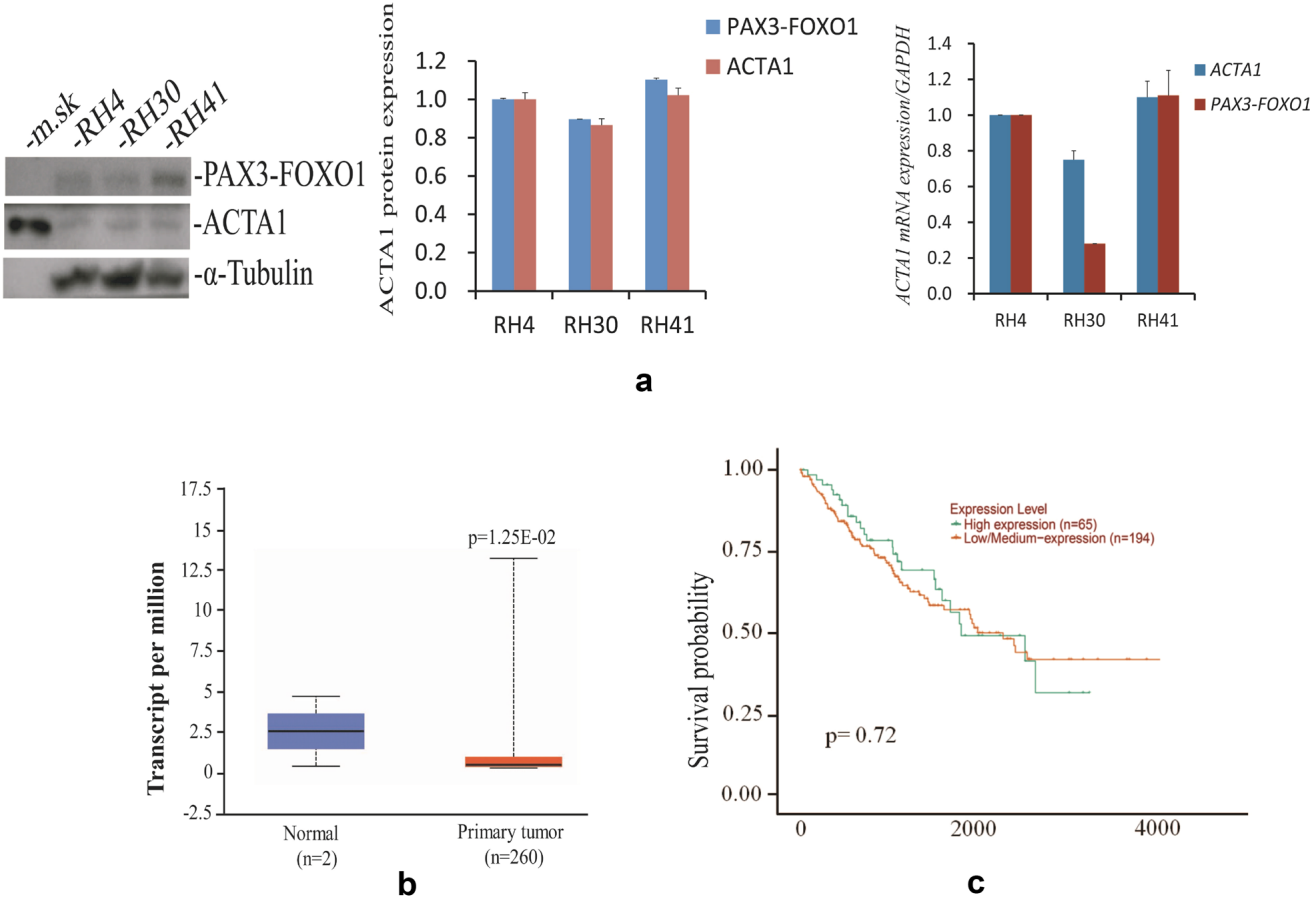
Protein expression of *ACTA1* gene in *Alveolar Rhabdomyosarcoma* (ARMS) cell lines. **a** Left: ACTA1 protein was examined by western blot in ARMS cell lines (RH30, RH4 and RH41). ACTA1 protein from mouse skeletal muscle tissue (*m.sk*) was shown as a control with same molecular weight as that from ARMS cells. α-tubulin is used as a loading control. Middle: Quantification of endogenous expression of PAX3-FOXO1 and ACTA1 proteins in cell lines normalized to α-tubulin and analyzed with ImageJ software. Right: Quantitative RT-PCR (qRT-PCR) analysis of *ACTA1* mRNA expression in ARMS cell lines. **b** *ACTA1* expression levels in normal vs primary sarcoma tumor samples from TCGA dataset (p = 1.25E-02). **c** Survival probability of sarcoma patients with low or high *ACTA1* expression (modified from online UALCAN)

*ACTA1* is a very conserved gene encoding a protein with high identical amino acid sequence from rice to human [13]. Although *ACTA1* transcript had been described in some RMS tumor samples [28], little work was paid attention to its protein expression and regulation in ARMS cells. By western blot analysis, we defined the expression of ACTA1 protein in ARMS cell lines and showed the difference among them to a certain extent (Fig. 1a, left and middle). ACTA1 protein appeared expressed at relative higher levels in RH4 and RH41 cells than that in RH30 cells with lower expression. mRNA levels determined by quantitative RT-PCR exhibited a similar expression pattern to that of ACTA1 proteins in ARMS cell lines (Fig. 1a, right). Due to the lack of information about *ACTA1* in *Rhabdomyosarcoma* in public database, we analyzed the expression of *ACTA1* in related *Sarcoma* which usually includes *Rhabdomyosarcoma*, *liposarcoma* and so on based on the TCGA dataset using the online UALCAN program (http://ualcan.path.uab.edu) [27]. As indicated in Fig. 1b, *ACTA1* transcript is significantly decreased in primary tumor compared to normal tissue (p = 1.25E-02), despite the low number of normal samples used. Obviously, patients with higher *ACTA1* expression displays a little increase in the 3- or 5-year survival probability compared to those with lower expression, even though the p-value (p = 0.72) shown in the graph is not significantly over the 10 years of time course (Fig. 1c). These analyses still suggested that there may be a certain link between ARMS and the lower *ACTA1* expression.

### *ACTA1* is inhibited by *PAX3-FOXO1* at transcriptional and translational levels in ARMS cells

**Fig. 2:**
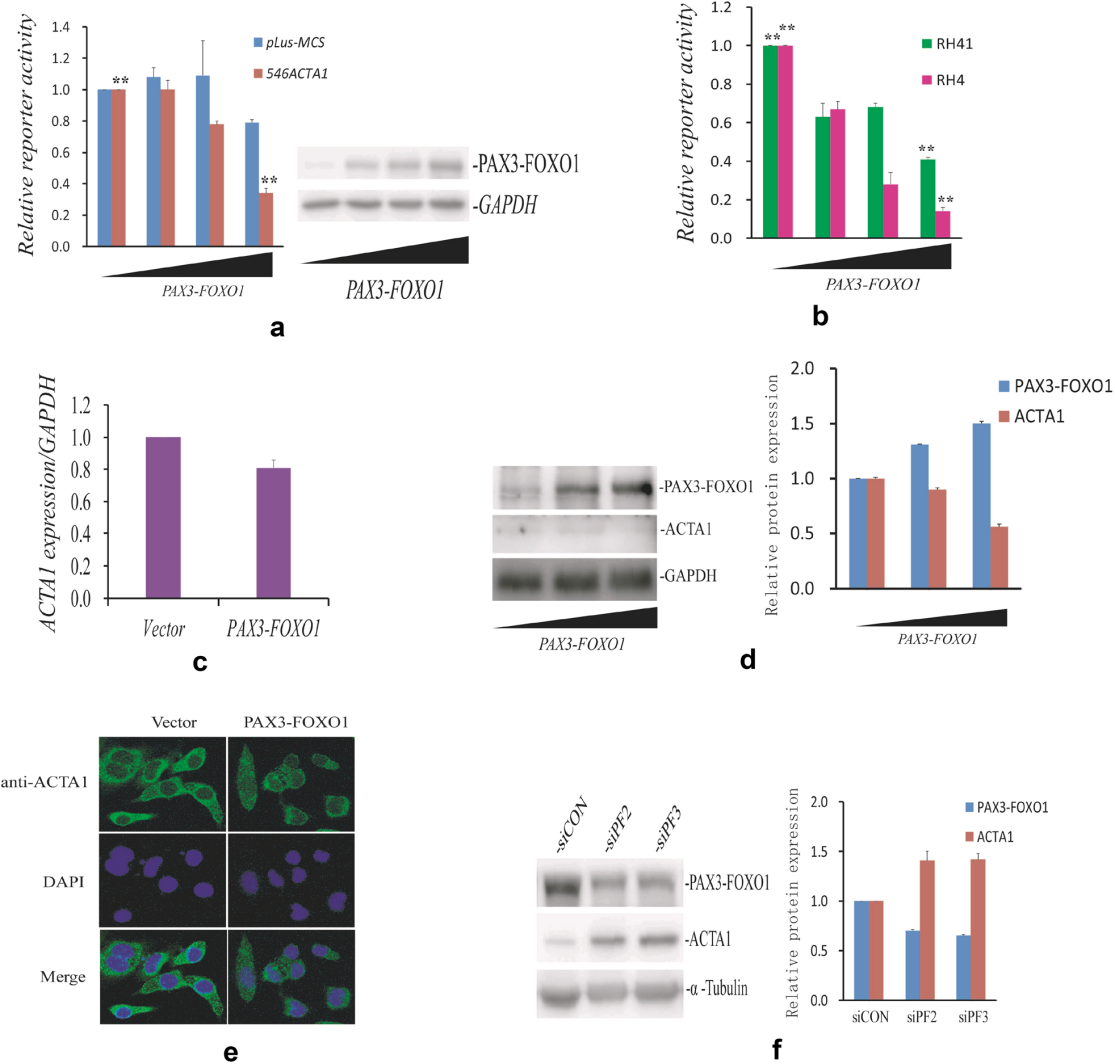
*ACTA1* is downregulated by *PAX3-FOXO1* fusion gene. **a** Luciferase activity of human *ACTA1* gene reporter (*546ACTA1*) is inhibited by PAX3-FOXO1 in RH30 cells. Left: *p*Luc-MCS or *546ACTA1* reporter cotransfected RH30 cells in 24-well plate with internal control  pRL-TK and increasing amounts of *PAX3-FOXO1* expression plasmid (0, 50, 100, 300 ng). Cells were maintained in DMEM-0.3% FBS-1% P/S for 24-36 h after transfection. Firefly luciferase activity was measured and normalized to Renilla luciferase activty. The reporter activity from the empty expression vector was set to one. Right: Representative assay of PAX3-FOXO1 protein levels by western blot in reporter transfections. **b** Luciferase activity of human *ACTA1* gene reporter (*546ACTA1*) was repressed by *PAX3-FOXO1* fusion gene in RH4 and RH41 cell lines. *546ACTA1* reporter was cotransfected with pRL-TK and increasing amounts of *PAX3-FOXO1* expression plasmid (0, 50, 100, 300 ng) into RH4 or RH41 cells. Assay of luciferase activity was performed as in (**a**). **c** mRNA expression of *ACTA1* in PAX3-FOXO1 overexpressed RH30 cells by qRT-PCR. *ACTA1* expression was normalized to human GAPDH levels. Bars represent the mean ± SEM. **d** ACTA1 protein expression in RH4 cells transfected with increasing amount of *PAX3-FOXO1* plasmid. Left: ACTA1 expression levels were assayed by western blot analysis. Right: the relative expression of ACTA1 protein shown on the left. **e**. Immunofluorescence analysis of ACTA1 expression in RH4 cells with overexpressed PAX3-FOXO1 protein. ACTA1 expression (green), detected to be primarily localized in cytoplasm, was obviously decreased in cells with ectopic PAX3-FOXO1 protein expression. **f** Knockdown of PAX3-FOXO1 by siRNA oligonucleotides (siPF2, siPF3) increased ACTA1 expression through western blotting. Left: Protein levels of ACTA1 and PAX3-FOXO1 expression assayed by western blot analysis. Right: the quantitative analyses of ACTA1 and PAX3-FOXO1 proteins shown on the left

*PAX3-FOXO1* is a specific fusion gene and codes for a transcription factor in  ARMS. To assess the possible regulation of *ACTA1* by *PAX3-FOXO1* in  ARMS cells, we cloned the promoter region of *ACTA1* into pLuc-MCS vector to obtain the human *ACTA1* reporter *(546ACTA1*) and cotransfected the RH30 cells, together with renilla luciferase control and increasing amounts of *PAX3-FOXO1* expression plasmid. Following 24 to 36 h serum free starvation, the luciferase activity of *ACTA1* reporter was measured using the dual-luciferase reporter system. The results indicated that *PAX3-FOXO1* decreased the *ACTA1* promoter activity in a dose dependent manner compared with the empty vector expression plasmid (Fig. 2a, left). In the same transfections of RH30 cells but replaced the *546ACTA1* reporter with pLuc-MCS control reporter plasmid, the luciferase activity was not any changed significantly by *PAX3-FOXO1* in comparison to the empty vector control (Fig. 2a, left). The protein levels of total PAX3-FOXO1 expressed were also detected by Western blot to be gradually increased in the RH30 transfections with different amount of *PAX3-FOXO1* plasmid (Fig. 2a, right). This inhibition of *ACTA1* reporter activity was also observed in other *Alveolar Rhabdomyosarcoma* cell lines such as RH41 and RH4 cells (Fig. 2b). Consistent with the reporter activity, the mRNA level of *ACTA1* in RH30 cells with overexpressed PAX3-FOXO1 protein was also shown to be decreased by qRT-PCR analysis (Fig. 2c).

To further describe this inhibition of *ACTA1* by the fusion gene *PAX3-FOXO1*, we transiently transfected RH4 cells with various amount of *PAX3-FOXO1* expression plasmid. The cells were serum free-starved for 48 h after transfection and harvested to detect the ACTA1 protein levels using western blot. As shown in Fig. 2d, *ACTA1* expression (at protein level) in RH4 cells were gradually decreased or inhibited with increasing amounts of *PAX3-FOXO1* expression plasmid used, suggesting that *PAX3-FOXO1* could negatively control the protein expression of *ACTA1* gene in RH4 cells. Meanwhile, the immunofluorescence analysis in RH4 cells more directly showed the reduction of *ACTA1* expression regulated by *PAX3-FOXO1* gene (Fig. 2e). In addition, we transfected RH4 cells by use of the siRNA duplex specifically against *PAX3-FOXO1* gene and measured the *ACTA1* expression. Data in Fig. 2f showed that knockdown of *PAX3-FOXO1* drastically enhances *ACTA1* expression at protein levels. These data above strongly demonstrated that *ACTA1* is inhibited by *PAX3-FOXO1* in the ARMS cells.

### *PAX3-FOXO1* downregulates RhoA-MKL1-SRF signaling pathway to inhibit *ACTA1* expression activity

**Fig. 3:**
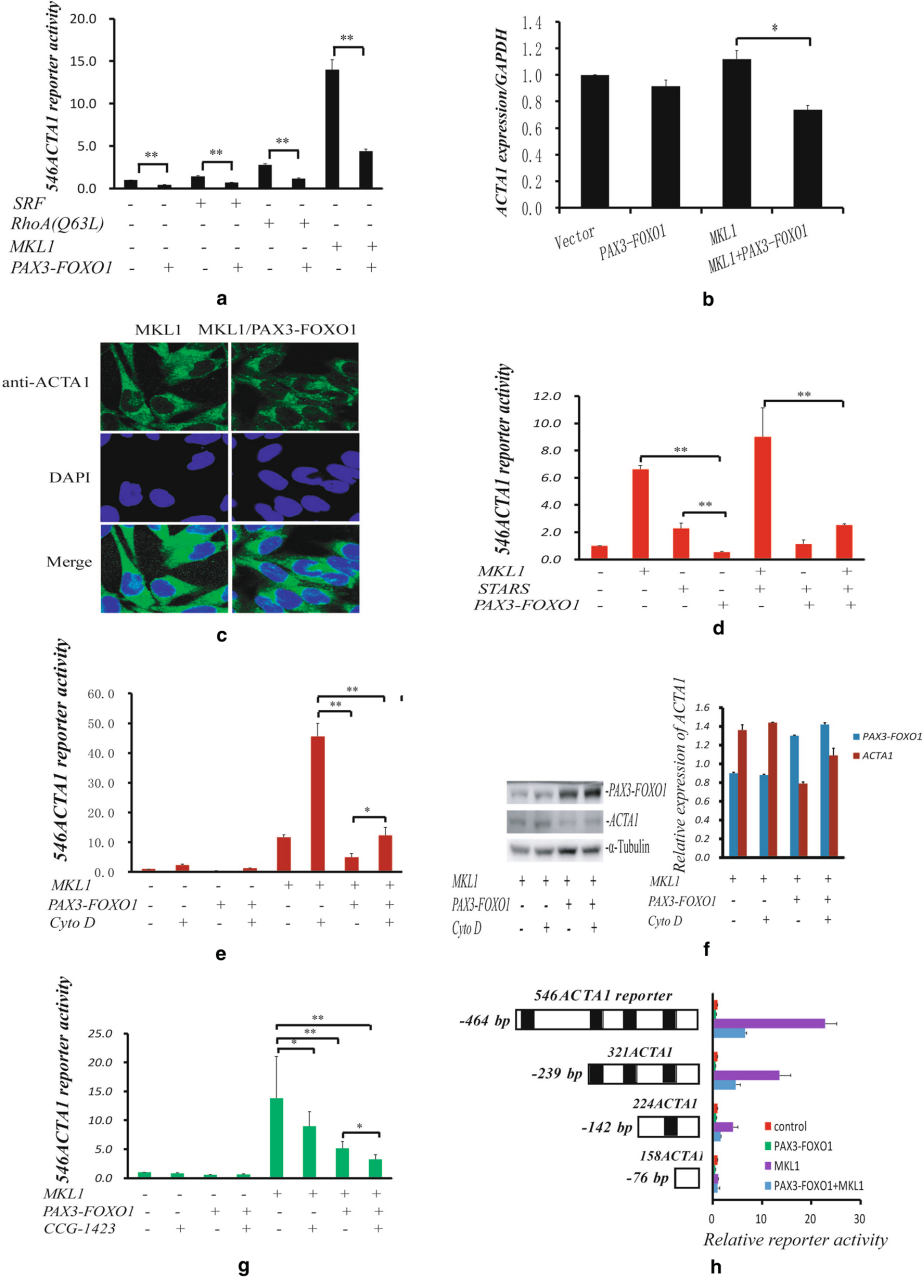
Downregulation of *ACTA1* by *PAX3-FOXO1* gene is associated with the repression of RhoA-MKL1-SRF signaling pathway. **a** Luciferase assays of RH30 cells transfected with *546ACTA1* reporter, along with pRL-TK, RhoA (Q63L), MKL1 or SRF in the presence or absence of PAX3-FOXO1 expression plasmid. **b**. qRT-PCR analysis of *ACTA1* in RH30 cells transfected with empty vector, MKL1 or MKL1 and *PAX3-FOXO1* plasmids together. **c** Immunofluorescence analysis of *ACTA1* expression using anti-ACTA1 specific antibody in RH30 cells transfected with MKL1 or MKL1 plus *PAX3-FOXO1* plasmid. DAPI was used to stain nucleus. ACTA1 protein level was tremendously decreased in cells with PAX3-FOXO1 overexpression. **d** Luciferase assays of RH30 cells cotransfected with *546ACTA1* reporter and pRL-TK, along with MKL1 and/or STARS in presence or absence of *PAX3-FOXO1* expression plasmid. **e** Luciferase assays of RH30 cells cotransfected with *546ACTA1* reporter and pRL-TK, along with MKL1 in the presence or absence of *PAX3-FOXO1* expression plasmid. In the DMEM with serum free starvation, cells were treated with 2 µM Cytochalasin D overnight as indicated and luciferase activities were then examined. **f** Analysis of ACTA1 protein expression modulated by Cytochalasin D. RH4 cells cotransfected with MKL1, or MKL1 and *PAX3-FOXO1* expression plasmid and treated with 2 µM Cytochalasin D were collected and lysed for the determination of ACTA1 expression. Left: Protein levels of ACTA1 and PAX3-FOXO1 assayed by western blot. Right: Relative expression of ACTA1 and PAX3-FOXO1 proteins shown on the left. **g** Luciferase assays of RH30 cells cotransfected with *546ACTA1* reporter and pRL-TK, along with empty vector, MKL1 and/or *PAX3-FOXO1* expression plasmid. As indicated, cells were treated with CCG-1423 at 10 uM for 5 h in the DMEM-0.3%FBS before luciferase assay. **h** Luciferase assays of RH30 cells cotransfected with *546ACTA1* wild type or CArG box-deleted mutant reporters, along with pRL-TK internal control, empty vector, MKL1 and/or *PAX3-FOXO1* expression plasmid. Black bar (■): CArG box

ACTA1 is a major actin isoform in skeletal muscle and it has been reported to be a target of serum response factor (SRF), a key protein in the RhoA-MKL1-SRF signaling pathway [19, 24]. Therefore, we investigated whether RhoA-MKL1-SRF signaling pathway is involved in *ACTA1* inhibition by *PAX3-FOXO1*. To this end, we used *546ACTA1* reporter to cotransfect the RH30 cells, together with MKL1, SRF or constitutive active mutant RhoA (Q63L) expression plasmid, in the presence of PAX3-FOXO1 protein expression and determined the luciferase activity under the same condition as in Fig. 2. In these transfected RH30 cells, all MKL1, SRF and RhoA (Q63L) could stimulate *ACTA1* reporter activities but these activities were reduced (at least twofold) by PAX3-FOXO1 expression (Fig. 3a). The strongly MKL1-stimulated mRNA level of *ACTA1* was accordingly decreased by *PAX3-FOXO1* using qRT-PCR analysis and the protein expression of *ACTA1* was also observed to be decreased by the immunofluorescence assay (Fig. 3b–c). STARS is an actin-binding protein that is specifically expressed in striated muscle to promote MKL1 nuclear accumulation and stimulate the transcriptional activity of SRF [22, 29], we evaluated whether *PAX3-FOXO1* could have some effect on the role of STARS in RH30 cells. As illustrated in Fig. 3d, the protein STARS could enhance the activity driven by MKL1 but this activity could be suppressed by PAX3-FOXO1 protein. Cytochalasin D (Cyto D) is a fungal metabolite to modulate actin dynamics and induce CTGF/CCN2 expression in tubular epithelial cells [30, 31], we therefore determined the behavior of Cyto D on *ACTA1* regulation in aRMS cells. As shown in Fig. 3e, Cyto D could induce the transcriptional activity of *ACTA1* reporter, but this induction was repressed by PAX3-FOXO1 expression. The ACTA1 protein was also examined and exhibited to be modulated by Cyto D in a similar manner of transcriptional activity in the presence of PAX3-FOXO1 protein (Fig. 3f). CCG-1423 has been a widely used inhibitor which can block MKL1 binding to importin α/ß1 protein and inhibit the RhoA-MKL1-SRF signaling pathway [32, 33], we next test whether *PAX3-FOXO1* could influence the action of CCG-1423 molecule. The luciferase activity showed the significant synergistic effect between *PAX3-FOXO1* and CCG-1423 (Fig. 3g) in the transfections of RH30 cells with co-expressed PAX3-FOXO1 and MKL1 proteins and followed by the treatment of CCG-1423. The similar synergistic effect between *PAX3-FOXO1* and CCG-1423 was also tested in RH41 cells (Additional file 1: Fig. S1). Finally, the analyses of a serial of deletion mutants created from the *ACTA1* promoter showed that the *ACTA1* activity regulated by *PAX3-FOXO1* was also CArG box dependent in the presence of MKL1 expression, implying the important association between *PAX3-FOXO1* and RhoA-MKL1-SRF pathway (Fig. 3h). Taken together, these results demonstrated that the RhoA-MKL1-SRF signaling pathway was involved in the function of *PAX3-FOXO1* to inhibit *ACTA1* expression.

### *PAX3-FOXO1* represses the total expression of MKL1 and SRF and thus their interaction but not affects their subcellular localization

**Fig. 4:**
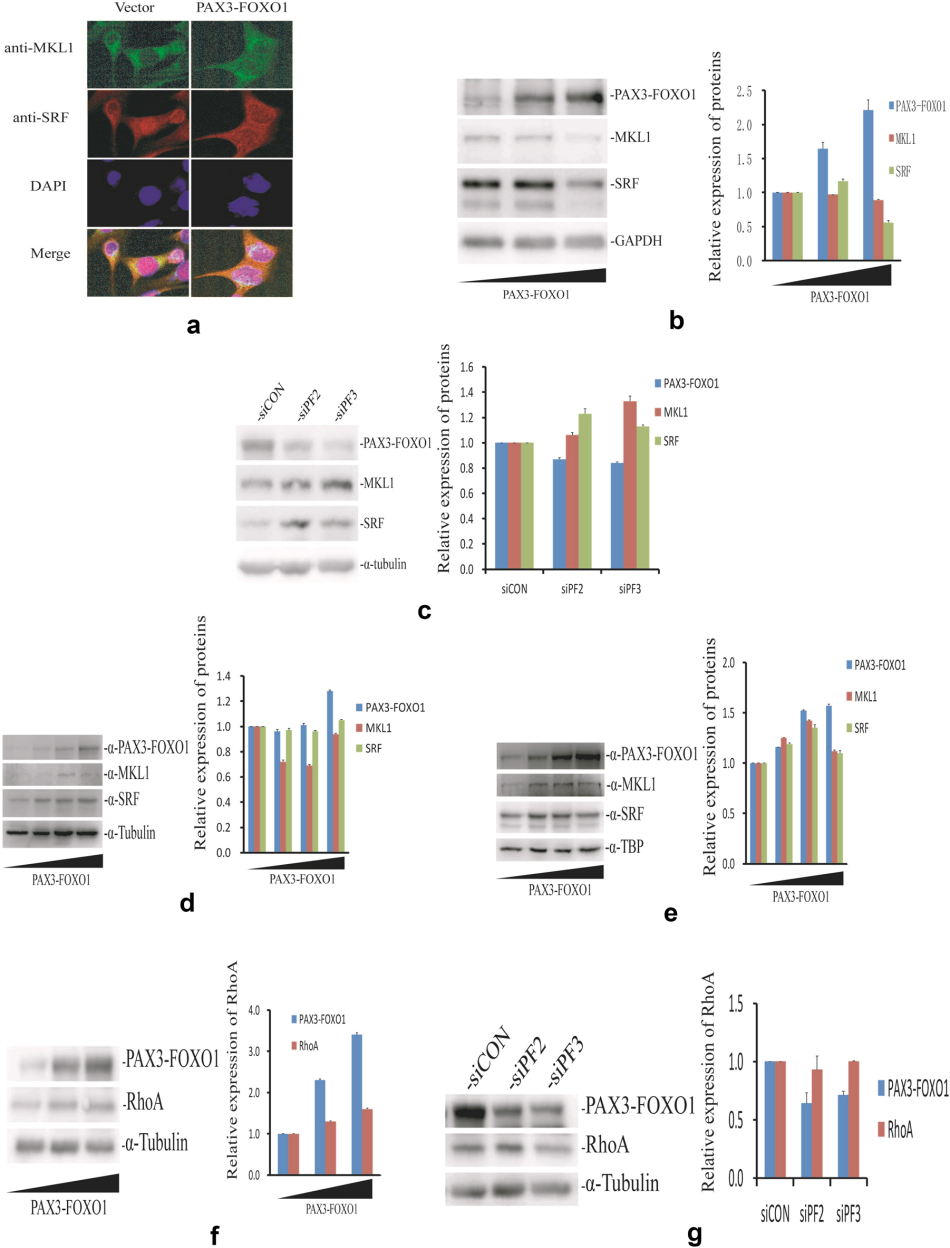
Expression and subcellular localization of MKL1 and SRF protein in ARMS cells. **a** Confocal images of MKL1 and SRF expression and subcellular localization in RH30 cells with or without overexpressed PAX3-FOXO1 protein. Protein expression of MKL1 (green) and SRF (red) and the colocalization or association (yellow) between them were attenuated with ectopic PAX3-FOXO1 expression, but almost not changed for their subcellular distribution by immunofluorescence staining. **b** Analysis of total MKL1 and SRF expression in RH4 cells with increasing amount of PAX3-FOXO1 protein expression. Left: MKL1 and SRF levels determined by western blot. Right: the relative expression of MKL1 and SRF protein shown on the left. **c** Analysis of total MKL1 and SRF expression in RH4 cells transfected by siRNAs against *PAX3-FOXO1* gene. Left: Expression of MKL1 and SRF determined by western blot. Right: the relative expression of MKL1 and SRF protein shown on the left. **d** Distribution of SRF and MKL1 proteins in cytoplasmic fraction of RH30 cells transfected with increasing amount of *PAX3-FOXO1* plasmid. Left: Expression of MKL1, SRF and PAX3-FOXO1 protein determined by western blot analysis. Right: the relative quantification analysis of MKL1, SRF and PAX3-FOXO1 protein shown on the left. α-Tubulin served as a loading control. **e** Distribution of SRF and MKL1 expression in nuclear fraction of RH30 cells transfected with increasing amount of *PAX3-FOXO1* plasmid. Left: Expression of MKL1, SRF and PAX3-FOXO1 protein determined by western blot. Right: the relative quantification analysis of MKL1, SRF and PAX3-FOXO1 protein shown on the left. TBP served as a loading control for nuclear fraction. **f** Analysis of total RhoA expression in RH4 cells with a different dose of *PAX3-FOXO1* plasmid transfected. Left: RhoA level determined by western blot. Right: the relative expression of RhoA protein shown on the left. **g** Analysis of total RhoA expression in RH4 cells transfected by siRNAs against *PAX3-FOXO1* gene. Left: Expression of RhoA determined by western blot. Right: the relative expression of RhoA protein shown on the left

It is well known that the association of MKL1 and SRF is required for the regulation of muscle-specific target genes controlled by SRF [34, 35]. To further understand the inhibition of *ACTA1* by *PAX3-FOXO1*, we investigated whether *PAX3-FOXO1* plays a role in the expression and association of MKL1 and SRF by immunofluorescence staining. We overexpressed PAX3-FOXO1 protein in RH30 cells and examined the expression and colocalization of MKL1 and SRF proteins under laser confocal microscopy. As shown in Fig. 4a, MKL1(green staining) and SRF (red staining) were expressed in both nucleus and cytoplasm and the colocalization or association (yellow staining) of MKL1 and SRF mainly took place in cytoplasm or around nucleus in these *PAX3-FOXO1* or control plasmid transfected cells. Importantly, the colocalization (yellow staining) between MKL1 and SRF was significantly reduced in cells with *PAX3-FOXO1* overexpression compared to control cells, indicating that the protein level of MKL1 or SRF might be affected by *PAX3-FOXO1*. We thus transfected *PAX3-FOXO1* gene into RH4 cells and determined the expression of MKL1 and SRF (Fig. 4b). The results in Fig. 4b indicated that *PAX3-FOXO1* can indeed repress the protein expressions of MKL1 and SRF. Corresponding to this data, the protein levels of MKL1 and SRF were strongly increased in the RH4 cells with *PAX3-FOXO1* knockdown using siRNAs (Fig. 4c). Meanwhile, we determined the subcellular localization of MKL1 and SRF proteins in cells with increasing amount of *PAX3-FOXO1* overexpression. The data in Fig. 4d, e demonstrated that *PAX3-FOXO1* had no any significant effect on the subcellular distribution of MKL1 and SRF proteins. The relative quantities of MKL1 and SRF expression in nuclear or cytoplasmic fraction were not significantly changed and both proteins were primarily localized in nucleus in cells with increasing *PAX3-FOXO1* overexpression. Additionally, the protein levels of RhoA were also measured in the case of overexpression or knockdown of *PAX3-FOXO1* and no obvious alteration of RhoA were detected (Fig. 4f, g). These results strongly demonstrated that the *ACTA1* inhibition by *PAX3-FOXO1* occurs through the repression of MKL1 and SRF, but not of RhoA expression in the RhoA-MKL1-SRF signaling pathway.

### Ectopic overexpression of *ACTA1* inhibits cell proliferation and cell migration

**Fig. 5:**
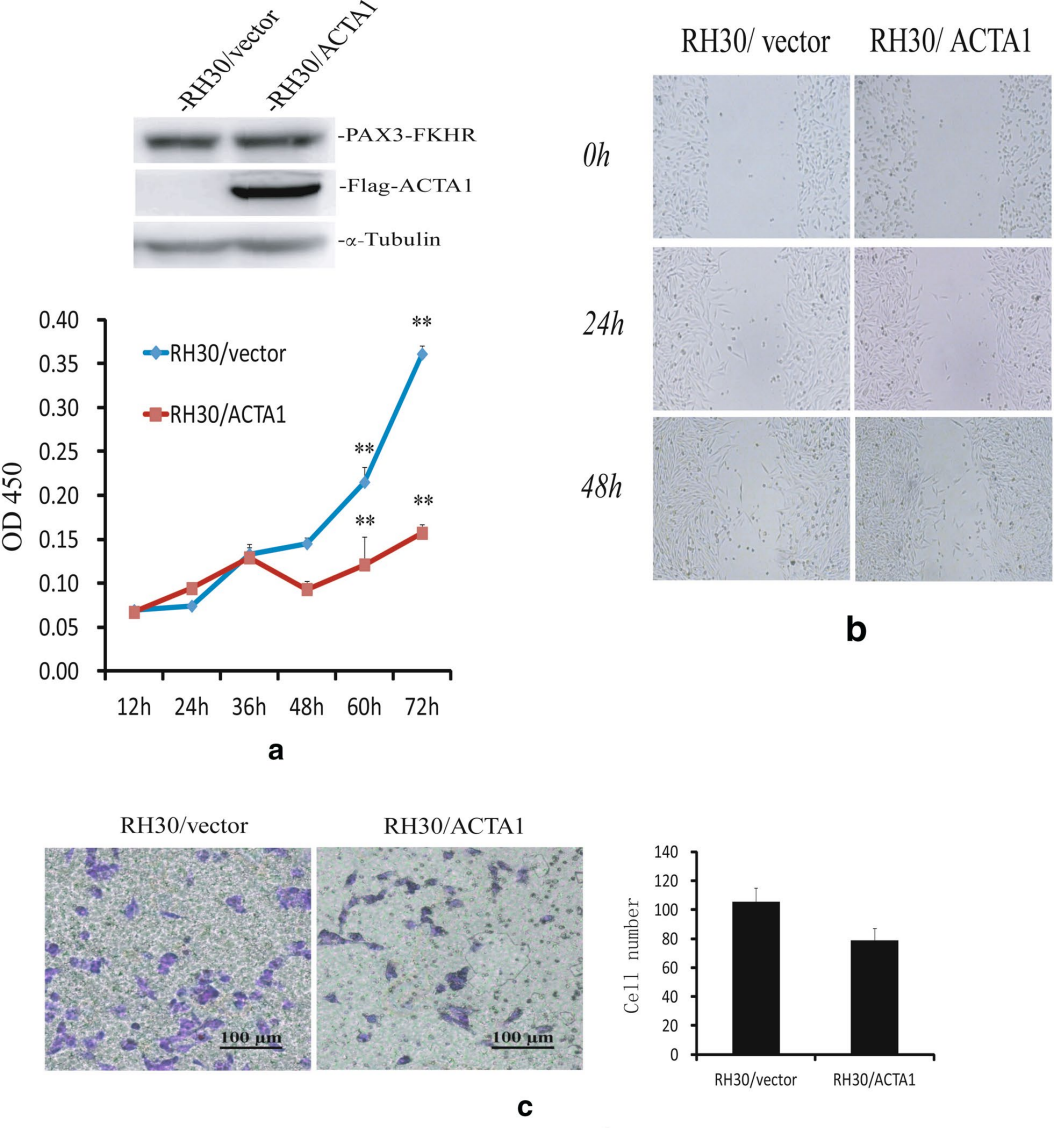
Effects of overexpressed ACTA1 on RH30 cell proliferation and migration. **a** Proliferation rate of stable cell lines RH30/vector and RH30/ACTA1 was determined by CCK-8 kit over 0-72 h (Lower panel) and assay of Flag-ACTA1expression in RH30/ACTA1 by western blot (Upper panel). **b** Wound healing assay was performed to detect the migration ability of the two cell lines RH30/vector and RH30/ACTA1. Upper panel: Representative images were taken at 0, 24 and 48 h. Lower panel: Relative migration rate of cells. *p < 0.05, compared with RH30/vector cells. **c** Transwell migration assay was performed in cell lines of RH30/vector and RH30/ACTA1, which showed that overexpressed ACTA1 could inhibit cell motility. *p < 0.05, compared with RH30/vector cells

As a major constituent of skeletal muscle, *ACTA1* plays important roles in cell contraction, motility, structure and morphologic change [14, 15, 18]. In order to explore the potential role of *ACTA1* in ARMS, we established stable cell lines RH30/*ACTA1* and RH30/vector with over- or normal expression level of ACTA1 protein, respectively. CCK-8 assay was employed to determine the effect of *ACTA1* on cell proliferation. And the results showed that *ACTA1* overexpression in RH30 cells (RH30/*ACTA1*) could significantly inhibit the cell proliferation after 60 h compared with the control cells (RH30/vector) (Fig. 5a). Considering this behavior of *ACTA1* to impair the cell proliferation rate, we next examine the effect of *ACTA1* on cell migration by the classic scratch wound healing and transwell methods. In the wound healing assay, we found that the *ACTA1* expression in RH30 cells had moderately decreased the migration ability in comparison to the control cells (Fig. 5b). In the transwell assay shown in Fig. 5c, the cell number on the lower surface of the filter was less for the *ACTA1* overexpressed RH30 cells than the vector control cells. These assays suggested that *ACTA1* overexpression could inhibit cell growth and reduce the cell migration ability. Altogether, these results shown above led us to further investigate the potential role of *ACTA1* in ARMS tumorigenesis.

### Overexpression of *ACTA1* suppresses tumor growth in nude mice

**Fig. 6:**
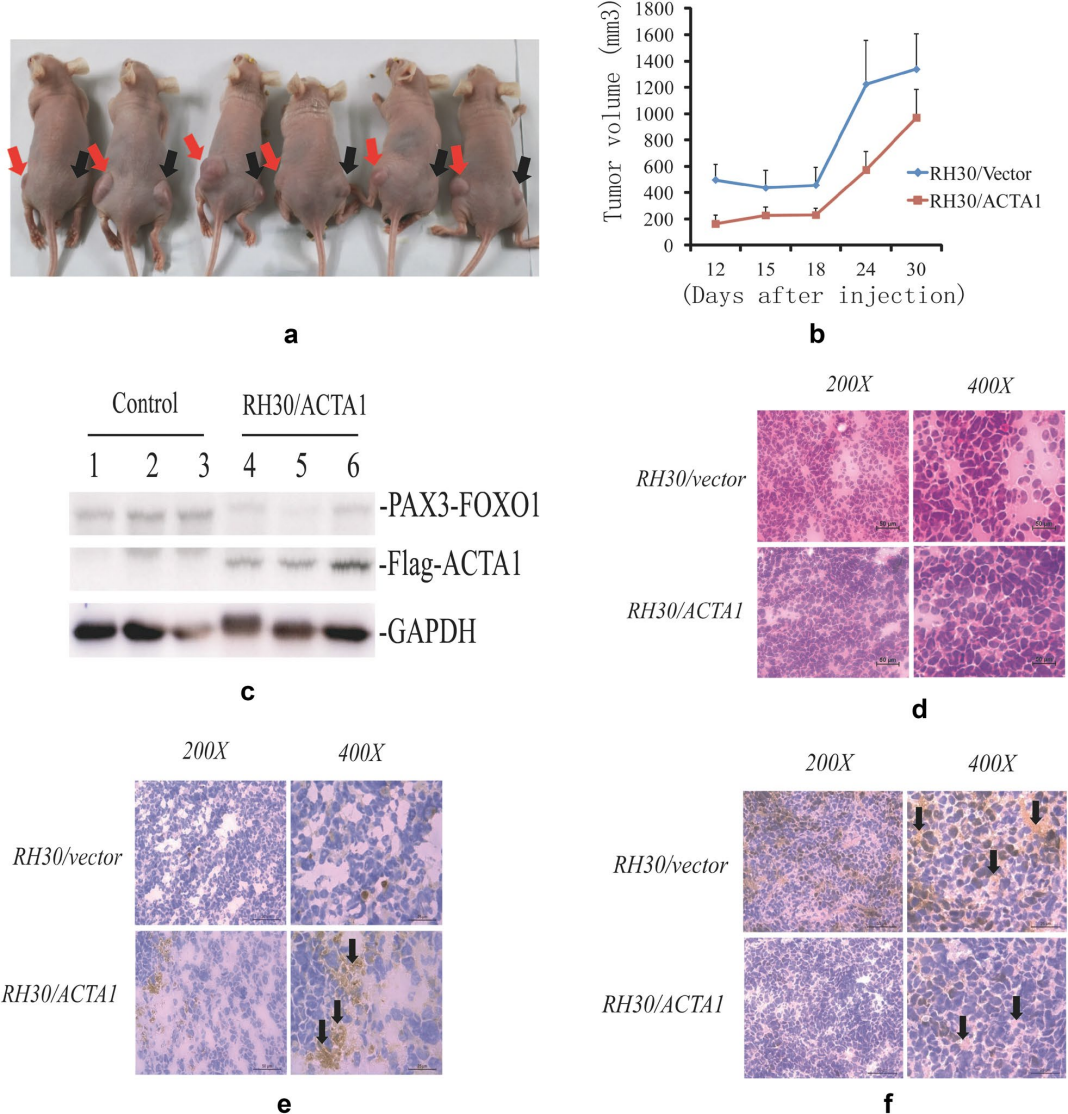
*ACTA1* overexpression suppresses tumor growth originated from RH30 cells in nude mice. **a** Photograph of tumor growth in representative nude mice. Left flank: injected with RH30/vector cells. Right flank: injected with RH30/*ACTA1* cells. Tumors in the left and right sides were indicated by red arrow and black arrow, respectively. **b** Growth curve of tumors originated from RH30/vector control and RH30/*ACTA1* cell lines. Data are from two independent experiments. Error bars, SEM. **c** Western blot analyses for the detection of PAX3-FOXO1 and ACTA1 (Flag-ACTA1) protein expression in 3 representative tumor samples originated from RH30/vector (1,2,3) and RH30/*ACTA1* (4,5,6) cell lines, respectively. Gapdh was used as a loading control. **d** H&E staining from representative tumor samples; **e** Flag staining for ACTA1 expression from representative tumor samples; **f** Staining for Ki67 expression from representative tumor samples. Arrow indicates ACTA1 (Flag-ACTA1) or Ki-67 expression. The expression level of Flag-ACTA1 or Ki-67 was judged with score 0 (negative), 1 (weak) and 2 (strong) based on the staining intensity and percentage of positive cells in the slide image

To evaluate the role of *ACTA1* in ARMS tumorigenesis, we established a xenograft model by s.c inoculating the empty vector and *ACTA1* overexpressed stable cell lines into the left and right hind limbs of male nude mice, respectively. 2 × 10^6^ cells in 200 ul PBS were used for each injection site. About 12 days after injection, palpable solid tumors were visible in the flanks of the mice. The tumor sizes were measured two times a week and the tumors were harvested about 5 weeks later of injection when the mice were sacrificed. The average size of the tumors resulting from RH30/*ACTA1* cell line was clearly smaller than that from the vector control cells (Fig. 6a). The Mean ± SEM tumor volume from the RH30/vector control cells was 1338.21 ± 267.1 mm^3^ at the end of the experiment. In contrast, the tumor volume from RH30/*ACTA1* cells was 969.41 ± 214.4 mm^3^, which suggested that the tumor growth may be suppressed by *ACTA1* overexpression (Fig. 6b). Meanwhile, these tumor samples were evaluated to be composed of a large amount of small round cells in the tissue section by H&E staining, showing the same characteristic as RMS tumor (Fig. 6d). In addition, overexpression of ACTA1 (Flag-ACTA1) protein was detected in tumors from RH30/*ACTA1* cells by western blot and immunohistochemistry analyses (Fig. 6c, e). We also detected the expression differences of Ki67 in the tumor section with the higher level of Ki67 expression in tumors derived from RH30/vector control cells than those from RH30/*ACTA1* (Fig. 6f), suggesting the inhibitory effect of *ACTA1*on tumor growth. Together, the data in the xenograft assay demonstrated that *ACTA1* overexpression could suppress tumor growth in nude mice and thus may play a role in the tumorigenesis or progression of aRMS in vivo.

## Discussion

RMS is thought to be associated with skeletal muscle tissue origin [2, 36]. However, the roles or functions of skeletal muscle proteins are less reported in RMS. ACTA1 is an important member of skeletal muscle proteins and is coexpressed with cardiac alpha-actin in adult skeletal muscle tissue [18]. Although early study showed the existence of the alpha skeletal muscle actin gene (*ACTA1*) transcript in ARMS tumor samples [28], no more detailed work about *ACTA1* in aRMS had been reported. In the present work, we firstly examined ACTA1 protein expression in ARMS cells by immunoblot and showed the possible association between *ACTA1* expression and ARMS (Fig. 1). Then we investigated the regulation of *ACTA1* expression in ARMS cells. To our surprise, we found that *ACTA1* could be a novel candidate target gene of *PAX3-FOXO1* based on its decreased expression at transcription and protein levels under the condition of overexpressed PAX3-FOXO1 protein. It has been well known that *ACTA1* is activated by SRF or its coactivator MKL1. We thus tested whether this decrease or inhibition of *ACTA1* expression by *PAX3-FOXO1* is associated with SRF or MKL1. To the end, we co-transfected RH30 cells with *ACTA1* gene reporter (*546ACTA1*), SRF, MKL1 and/or *PAX3-FOXO1* expression plasmids and measured the luciferase activity. As expected, these activities were repressed by *PAX3-FOXO1* in comparison to those induced by SRF or MKL1 protein. We also tested the effects of RhoA and STARS on the *ACTA1* promoter activity and obtained the results similar to those from MKL1 and SRF in the presence of PAX3-FOXO1 overexpression. In addition, the analysis of the functional site within the promoter region revealed that this inhibition of *ACTA1* transcription activity was CArG box dependent (Fig. 3h), demonstrating that the inhibitory action by fusion gene *PAX3-FOXO1* is closely related to the RhoA-MKL1-SRF signaling pathway. CCG-1423 is an inhibitor of the RhoA-MKL1-SRF signaling pathway [37], the strongly synergistic effect between *PAX3-FOXO1* and CCG-1423 further established the specific function of *PAX3-FOXO1* in *ACTA1* regulation in ARMS cells. Furthermore, the *ACTA1* activity exerted by Cytochalasin D could also be blocked by *PAX3-FOXO1* in these cells. Finally, the measurement of mRNA and protein levels of *ACTA1* expression directly showed the inhibitory role by fusion gene *PAX3-FOXO1* in ARMS cells. From these results, we concluded that *ACTA1* expression is inhibited or downregulated by *PAX3-FOXO1* through the RhoA-MKL1-SRF signaling pathway in ARMS cells.

Many target genes regulated by the transcription factor PAX3-FOXO1 fusion protein have been identified so far [38–41], but genes that were inhibited by PAX3-FOXO1 were relatively less reported or not fully explored in RMS cell system. To address whether the skeletal muscle alpha actin gene *ACTA1,* the newly identified candidate target of *PAX3-FOXO1* plays a role in ARMS, we created the stable cell line (RH30 /*ACTA1*) with *ACTA1* overexpression and evaluated the effect of *ACTA1* on ARMS cells. We observed that *ACTA1* overexpression could obviously impair the proliferation rate of RH30 cells when compared to the control cells (RH30 /vector). The scratch wound healing assay showed the RH30 /*ACTA1* cells migrated slower than the control cells. Finally, the xenograft assay demonstrated that *ACTA1* overexpression could suppress the tumor growth in the mouse model system used. These results are almost consistent with the observations from *PAX3-FOXO1* knockdown by Kikuchi et al. [12], suggesting that *ACTA1* might play an important role in ARMS tumorigenesis or development. However, the colony formation assay in our experiment didn’t show the inhibitory effect of *ACTA1* to cell proliferation (Additional file 1: Fig. S2.). It may be the presence of other actin isoforms that obscure the behavior of ACTA1 in ARMS cells during the longer period of incubation. Whether this implies that ACTA1 may also be associated with apoptosis is unknown. Therefore, it will be interesting to determine these assays in future by use of other ARMS cells with *ACTA1* expression knockdown or knockout.

**Fig. 7:**
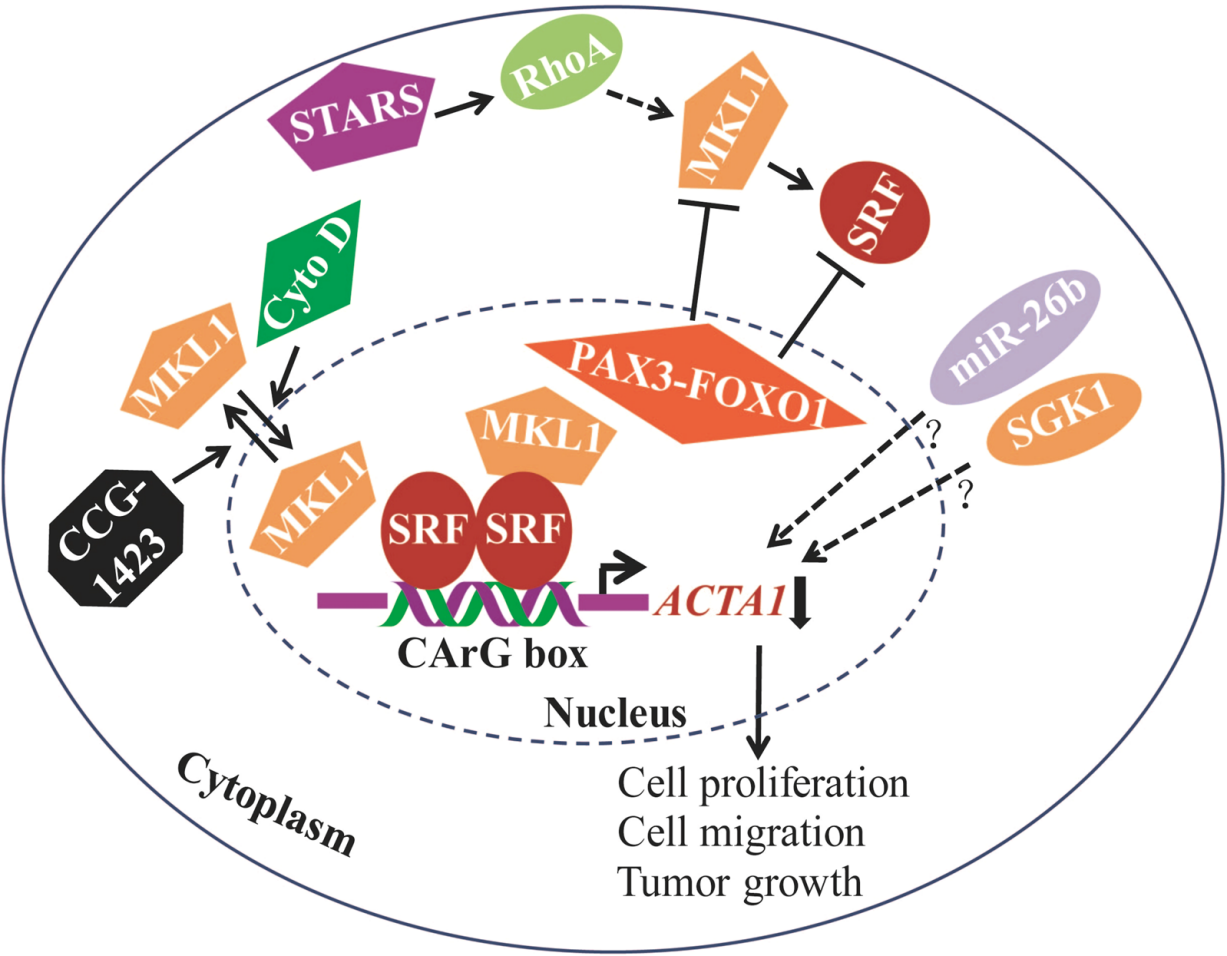
Proposed model of *ACTA1* inhibition by *PAX3-FOXO1* in ARMS cells. *ACTA1* is inhibited by *PAX3-FOXO1* through RhoA-MKL1-SRF signaling pathway. In this pathway, Cyto D and CCG-1423 can regulate *ACTA1* expression by controlling nuclear accumulation of MKL1 in distinct ways. *PAX3-FOXO1* can cooperate with these molecules in *ACTA1* regulation. SRF activates *ACTA1* expression by binding to the promoter region of *ACTA1* gene. Overexpressed ACTA1 protein can inhibit ARMS cell proliferation, migration and tumor growth. Therefore, decreased *ACTA1* expression by *PAX3-FOXO1* may help to promote cell proliferation, migration and finally tumor growth.

*ACTA1* expression in cells is complicated and regulated by multiple ways. In cardiomyocytes, *ACTA1* transcription is upregulated by serum- and glucocorticoid-inducible kinase (SGK1) and Small CTD phosphatases (SCP1) protein [42, 43]. miRNA-26b and *Myolinc* have a negative or positive role on *ACTA1* expression in cardiomycytes and myogenesis, respectively [43, 44]. All these studies demonstrated the complexity and importance of *ACTA1* expression and regulation in cells. The mechanism analysis in our work further revealed the similar and remarkable inhibition of MKL1 and SRF expression by *PAX3-FOXO1* in RH4 cells, but without alteration in the nuclear accumulation of MKL1 and SRF proteins in RH30 ARMS cells. Meanwhile, RhoA expression in the same signaling pathway was almost not affected by *PAX3-FOXO1*. We therefore supposed that *ACTA1* inhibition by *PAX3-FOXO1* could be as a result of the repression of MKL1 and/or SRF expression in ARMS cells. A postulated model describing the *ACTA1* inhibition by *PAX3-FOXO1* and eventually leading to cell and tumor growth is shown in Fig. 7. To our knowledge, this is the first report about the expression and regulation of *ACTA1* by *PAX3-FOXO1* in ARMS cells. Obviously, these findings are helpful to further understand the involvement of *PAX3-FOXO1* in ARMS tumorigenesis. This study also suggested that the RhoA-MKL1-SRF signaling pathway may play an important role in ARMS disease. In future work, the detailed process of *PAX3-FOXO1* to repress MKL1 or SRF will be explored. And other effects of *ACTA1* expression or of the novel RhoA-MKL1-SRF signaling pathway on the tumorigenesis of ARMS will be further studied at the same time.

## Conclusions

We investigated *ACTA1* expression and identified that *ACTA1* was inhibited by *PAX3-FOXO1* fusion gene at mRNA and protein levels in ARMS cells. The mechanism underlying this inhibition was further revealed to be involved in the RhoA-MKL1-SRF signaling pathway with repressions of MKL1 and SRF but not RhoA expression by *PAX3-FOXO1*. In addition, the distribution of MKL1 and SRF in nucleus or cytoplasm was demonstrated not to be significantly changed by *PAX3-FOXO1* expression. The potential role of *ACTA1* to impair cell and tumor growth in ARMS was explored by in vitro and in vivo experiments. Therefore, *ACTA1* inhibition by *PAX3-FOXO1* may play an important role in the development of ARMS. And the appropriate control of *ACTA1* expression and/or RhoA-MKL1-SRF signaling pathway might be a potential strategy for ARMS treatment in future.

## Supplementary Information

**Additional file 1: Figure S1.** Synergistic effect between *PAX3-FOXO1* and CCG-1423 to inhibit ACTA1 activity. **Figure S2.** Cell colony formation assay.

## Data Availability

All data in this study are included in this manuscript.

## Abbreviations

ARMS: *Alveolar Rhabdomyosarcoma*
*ACTA1*: Skeletal muscle alpha actin gene
*PAX3-FOXO1*: *PAX3-FOXO1* Fusion gene
G-actin: Globular actin
F-actin: Filamentous actin
FBS: Fetal bovine serum
P/S: Penicillin/streptomycin
qRT-PCR: Quantitative real-time polymerase chain reaction
SRF: Serum response factor
MKL1/ MRTF-A: Megakaryoblastic leukemia 1/myocardin related transcription factor-A
RhoA: Ras homolog gene family, member A
Cyto D: Cytochalasin D
PFA: Paraformaldehyde
H&E: Hematoxylin and eosin

## References

[1] Dasgupta R, Fuchs J, Rodeberg D (2016). Rhabdomyosarcoma. Semin Pediatr Surg.

[2] Lagutina IV, Valentine V, Picchione F, Harwood F, Valentine MB, Villarejo-Balcells B (2015). Modeling of the human alveolar rhabdomyosarcoma Pax3-Foxo1 chromosome translocation in mouse myoblasts using CRISPR-Cas9 nuclease. PLoS Genet.

[3] Kasiappan R, Jutooru I, Mohankumar K, Karki K, Lacey A, Safe S (2019). Reactive Oxygen Species (ROS)-Inducing Triterpenoid Inhibits Rhabdomyosarcoma Cell and Tumor Growth through Targeting Sp Transcription Factors. Mol Cancer Res.

[4] Dionyssiou MG, Ehyai S, Avrutin E, Connor MK, McDermott JC (2014). Glycogen synthase kinase 3b represses MYOGENIN function in alveolar rhabdomyosarcoma. Cell Death and Disease.

[5] Davis RJ, Barr FG (1997). Fusion genes resulting from alternative chromosomal translocations are overexpressed by gene-specific mechanisms in alveolar rhabdomyosarcoma. Proc Natl Acad Sci USA.

[6] Oh TJ, Adhikari A, Mohamad T, Althobaiti A, Davie J 2019. TBX3 represses TBX2 under the control of the PRC2 complex in skeletal muscle and rhabdomyosarcoma**.** Oncogenesis. 12;8(4):27. 10.1038/s41389-019-0137-z. 10.1038/s41389-019-0137-z PMC6461654 30979887

[7] Davicioni E, Finckenstein FG, Shahbazian V, Buckley JD, Triche TJ, Anderson MJ (2006). Identification of a PAX-FKHR gene expression signature that defines molecular classes, determines the prognosis of alveolar rhabdomyosarcomas. Cancer Res.

[8] Nguyen TH, Barr FG, (2018). Therapeutic Approaches Targeting PAX3-FOXO1 and Its Regulatory and Transcriptional Pathways in Rhabdomyosarcoma. *Molecules.* 23(11). pii: E2798. doi: 10.3390/molecules23112798. 10.3390/molecules23112798 PMC6278278 30373318

[9] Bailey KA, Wexler LH (2020). Pediatric rhabdomyosarcoma with bone marrow metastasis. Pediatr Blood Cancer.

[10] Scheidler S, Fredericks WJ, Rauscher FJ, Barr FG, Vogt PK (1996). The hybrid PAX3-FKHR fusion protein of alveolar rhabdomyosarcoma transforms fibroblasts in culture. Proc Natl Acad Sci USA.

[11] Xia SJ, Holder DD, Pawel BR, Zhang C, Barr FG (2009). High expression of the PAX3–FKHR oncoprotein is required to promote tumorigenesis of human myoblasts. Am J Pathol.

[12] Kikuchi K, Tsuchiya K, Otabe O, Gotoh T, Tamura S, Katsumi Y (2008). Effects of PAX3-FKHR on malignant phenotypes in alveolar rhabdomyosarcoma. Biochem Biophys Res Commun.

[13] Laing NG, Dye DE, Wallgren-Pettersson C, Richard G, Monnier N, Lillis S (2009). Mutations and polymorphisms of the skeletal muscle alpha-actin gene (ACTA1). Hum Mutat.

[14] Nowak KJ, Ravenscroft G, Jackaman C, Filipovska A, Davies SM, Lim EM (2009). Rescue of skeletal muscle alpha-actin-null mice by cardiac (fetal) alpha-actin. J Cell Biol.

[15] Crawford K, Flick R, Close L, Shelly D, Paul R, Bove K (2002). Mice Lacking Skeletal Muscle Actin Show Reduced Muscle Strength and Growth Deficits and Die during the Neonatal Period. Mol Cell Biol.

[16] Chaponnier C, Gabbiani G (2004). Pathological situations characterized by altered actin isoform expression. J Pathol.

[17] Ilkovski B, Clement S, Sewry C, North KN, Cooper ST (2005). Defining alpha-skeletal and alpha-cardiac actin expression in human heart and skeletal muscle explains the absence of cardiac involvement in ACTA1 nemaline myopathy. Neuromuscul Disord.

[18] Tondeleir D, Vandamme D, Vandekerckhove J, Ampe C, Lambrechts A (2009). Actin isoform expression patterns during mammalian development and in pathology: insights from mouse models. Cell Motil Cytoskeleton.

[19] Lee TC, Chow KL, Fang P, Schwartz RJ (1991). Activation of skeletal alpha-actin gene transcription: the cooperative formation of serum response factor-binding complexes over positive cis-acting promoter serum response elements displaces a negative-acting nuclear factor enriched in replicating myoblasts and nonmyogenic cells. Mol Cell Biol.

[20] Chang PS, Li L, McAnally J, Olson EN (2001). Muscle specificity encoded by specific serum response factor-binding sites. J Biol Chem.

[21] Posern G, Treisman R (2006). Actin' together: serum response factor, its cofactors and the link to signal transduction. Trends Cell Biol.

[22] Kuwahara K, Barrientos T, Pipes GC, Li S, Olson EN (2005). Muscle-specific signaling mechanism that links actin dynamics to response factor. Mol Cell Biol.

[23] Hipp L, Beer J, Kuchler O, Reisser M, Sinske D, Michaelis J (2019). Single-molecule imaging of the transcription factor SRF reveals prolonged chromatin-binding kinetics upon cell stimulation. Proc Natl Acad Sci USA.

[24] Collard L, Herledan G, Pincini A, Guerci A, Randrianarison-Huetz V, Sotiropoulos A (2014). Nuclear actin and myocardin-related transcription factors control disuse muscle atrophy through regulation of Srf activity. J Cell Sci.

[25] Laing NG, Clarke NF, Dye DE, Liyanage K, Walker KR, Kobayashi Y (2004). Actin mutations are one cause of congenital fibre type disproportion. Ann Neurol.

[26] Hu Q, Yuan Y, Wang C (2013). Structural and functional studies of FKHR-PAX3, a reciprocal fusion gene of the t(2;13) chromosomal translocation in alveolar rhabdomyosarcoma. PLoS ONE.

[27] Chandrashekar DS, Bashel B, Balasubramanya SAH, Creighton CJ, Ponce-Rodriguez I, Chakravarthi B (2017). UALCAN: a portal for facilitating tumor subgroup gene expression and survival analyses. Neoplasia.

[28] Tonin PN, Scrable H, Shimada H, Cavenee WK (1991). Muscle-specific gene expression in rhabdomyosarcomas and stages of human fetal skeletal muscle development. Cancer Res.

[29] Arai A, Spencer JA, Olson EN (2002). STARS, a striated muscle activator of Rho signaling and serum response factor-dependent transcription. J Biol Chem.

[30] Huang FY, Mei WL, Li YN, Tan GH, Dai HF, Guo JL (2012). The antitumour activities induced by pegylated liposomal cytochalasin D in murine models. Eur J Cancer.

[31] Muehlich S, Rehm M, Ebenau A, Goppelt-Struebe M (2017). Synergistic induction of CTGF by cytochalasin D and TGFβ-1 in primary human renal epithelial cells: Role of transcriptional regulators MKL1, YAP/TAZ and Smad2/3. Cell Signal.

[32] Hayashi K, Watanabe B, Nakagawa Y, Minami S, Morita T (2014). RPEL proteins are the molecular targets for CCG-1423, an inhibitor of Rho signaling. PLoS ONE.

[33] Evelyn CR, Wade SM, Wang Q, Wu M, Iñiguez-Lluhí JA, Merajver SD (2007). CCG-1423: a small-molecule inhibitor of RhoA transcriptional signaling. Mol Cancer Ther.

[34] Selvaraj A, Prywes R (2003). Megakaryoblastic leukemia-1/2, a transcriptional co-activator of serum response factor, is required for skeletal myogenic differentiation. J Biol Chem.

[35] Zaromytidou AI, Miralles F, Treisman R (2006). MAL and ternary complex factor use different mechanisms to contact a common surface on the serum response factor DNA-binding domain. Mol Cell Biol.

[36] El Demellawy D, McGowan-Jordan J, de Nanassy J, Chernetsova E, Nasr A (2017). Update on molecular findings in rhabdomyosarcoma. Pathology.

[37] Bell JL, Haak AJ, Wade SM, Sun Y, Neubig RR, Larsen SD (2013). Design and synthesis of tag-free photoprobes for the identification of the molecular target for CCG-1423, a novel inhibitor of the Rho/MKL1/SRF signaling pathway. Beilstein J Org Chem.

[38] Ginsberg JP, Davis RJ, Bennicelli JL, Nauta LE, Barr FG (1998). Up-regulation of MET but not neural cell adhesion molecule expression by the PAX3-FKHR fusion protein in alveolar rhabdomyosarcoma. Cancer Res.

[39] Khan J, Bittner ML, Saal LH, Teichmann U, Azorsa DO, Gooden GC (1999). cDNA microarrays detect activation of a myogenic transcription program by the PAX3-FKHR fusion oncogene. Proc Natl Acad Sci U S A.

[40] Taulli R, Scuoppo C, Bersani F, Accornero P, Forni PE, Miretti S (2006). Validation of met as a therapeutic target in alveolar and embryonal rhabdomyosarcoma. Cancer Res.

[41] Liu L, Wang YD, Wu J, Cui J, Chen T (2012). Carnitine palmitoyltransferase 1A (CPT1A): a transcriptional target of PAX3-FKHR and mediates PAX3-FKHR-dependent motility in alveolar rhabdomyosarcoma cells. BMC Cancer.

[42] Voelkl J, Castor T, Musculus K, Viereck R, Mia S, Feger M (2015). SGK 1-Sensitive Regulation of Cyclin-Dependent Kinase Inhibitor 1B (p27) in Cardiomyocyte Hypertrophy. Cell Physiol Biochem.

[43] Sowa N, Horie T, Kuwabara Y, Baba O, Watanabe S, Nishi H (2012). MicroRNA 26b encoded by the intron of small CTD phosphatase (SCP) 1 has an antagonistic effect on its host gene. J Cell Biochem.

[44] Militello G, Hosen MR, Ponomareva Y, Gellert P, Weirick T, John D (2018). A novel long non-coding RNA Myolinc regulates myogenesis through TDP-43 and Filip1. J Mol Cell Biol.

