# Supplementary material for: ACTA1 is inhibited by PAX3-FOXO1 through RhoA-MKL1-SRF signaling pathway and impairs cell proliferation, migration and tumor growth in Alveolar Rhabdomyosarcoma
Source: Cell Biosci. 2021 Jan 28;11:25. doi: 10.1186/s13578-021-00534-3 (PMC7842031; doi:10.1186/s13578-021-00534-3)

Supplementary Figure 1:

**Synergistic effect between *PAX3-FOXO1* and CCG-1423 to inhibit ACTA1 activity.** Luciferase assay of *546ACTA1* reporter activity was performed using RH41 cells cotransfected with *546ACTA1* reporter and pRL-TK, along with empty vector, MKL1 and/or *PAX3-FOXO1* expression plasmid. As indicated, cells were treated with CCG-1423 at 10 uM for 5h in the DMEM-0.3%FBS before luciferase assay.


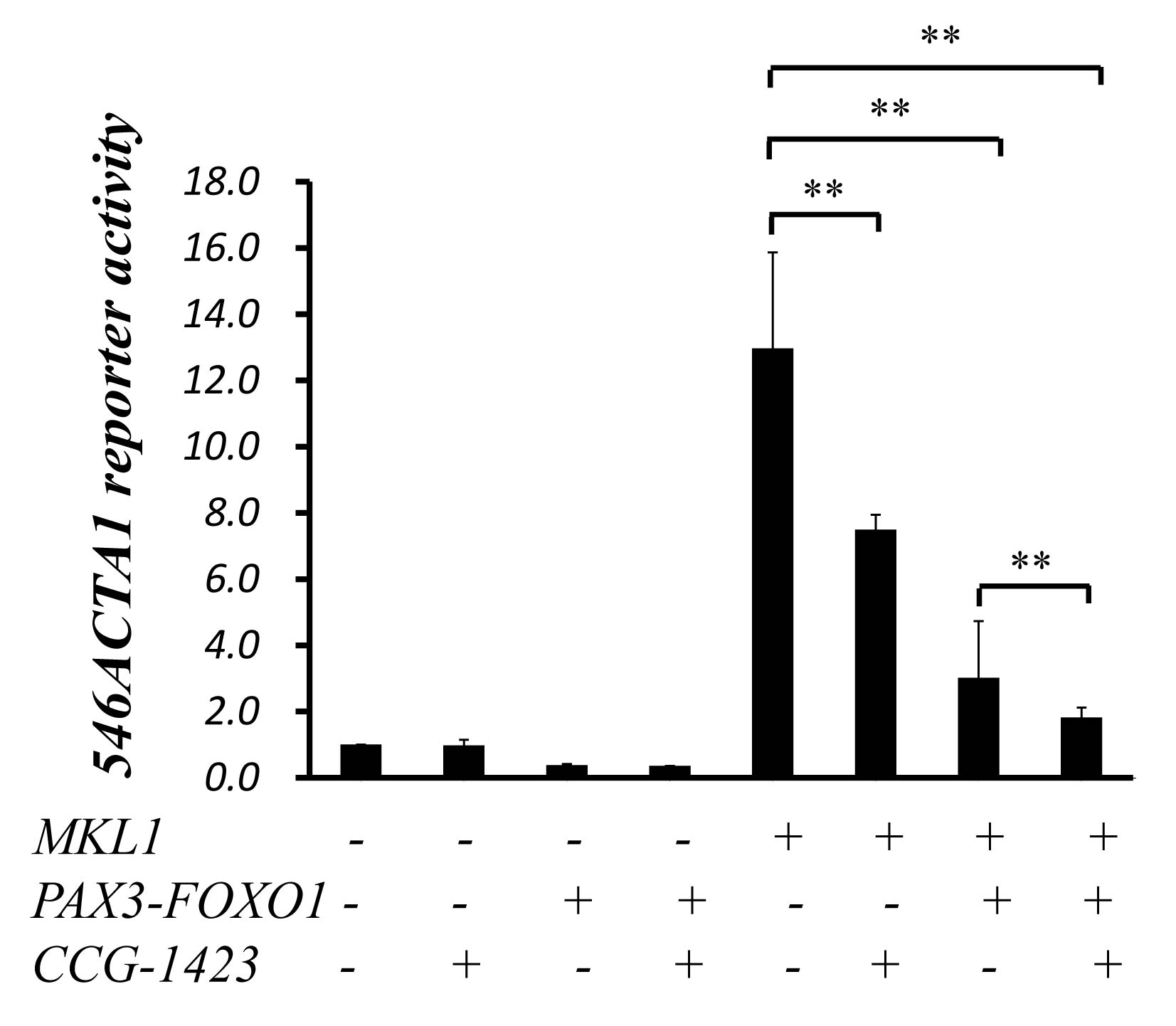


Supplementary Figure 2:

**Cell colony formation assay** 500 cells of RH30/*ACTA1* or vector control stable cell line were plated into 6-well in triplicate in DMEM-10%FBS-1%P/S and incubated for two weeks. The media were changed only one time at the end of the first week during the incubation. The cells were fixed by 4% paraformaldehyde (PFA) after washed with PBS and stained with 0.3% solution of filtered crystal violet. The colonies with diameters of more than 1.0 mm were manually counted and imaged but didn’t show the inhibitory effect of ACTA1 to the cell proliferation as expected (p > 0.05).


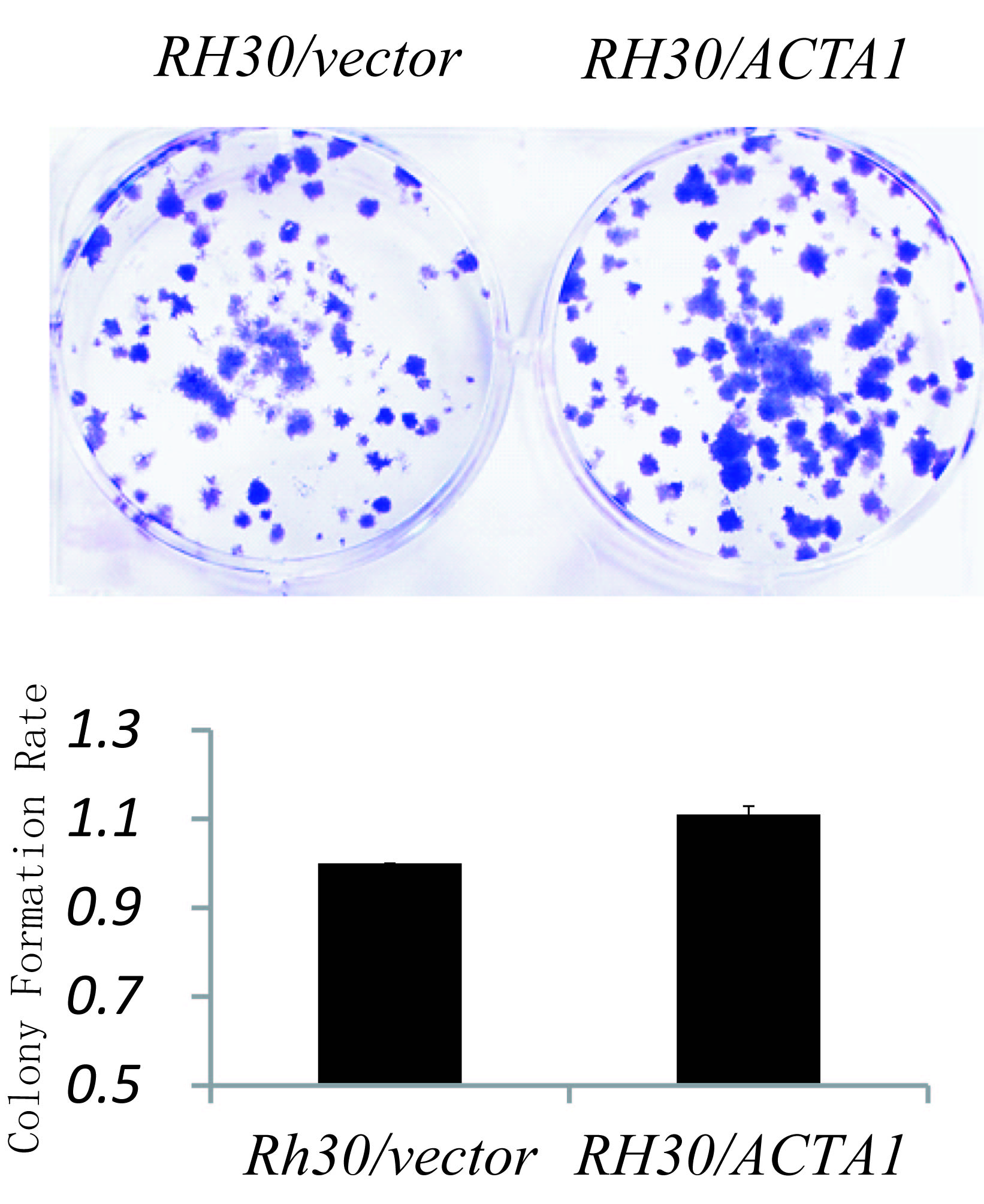

Supplement: Supplementary file 1 — Additional file 1: Figure S1. Synergistic effect between PAX3-FOXO1 and CCG-1423 to inhibit ACTA1 activity. Figure S2. Cell colony formation assay. [file 13578_2021_534_MOESM1_ESM.doc]
